# “You don’t treat your skinny patients like this”: a qualitative study of fertility care experiences among women with larger bodies and infertility

**DOI:** 10.3389/frph.2026.1730572

**Published:** 2026-02-04

**Authors:** Vanessa Elliott, Kaitlyn Plummer, Daphna Stroumsa, Ashley Hesson, Erica E. Marsh, Samantha B. Schon

**Affiliations:** 1 University of Michigan Medical School, Ann Arbor MI, United States; 2 Department of Obstetrics and Gynecology, University of Michigan, Ann Arbor, MI, United States

**Keywords:** fertility, infertility, obesity, qualitative, stigma

## Abstract

**Background:**

Prior studies show that people with larger bodies experience pervasive weight stigma, which is often directly perpetuated by healthcare providers. This pattern has also been observed in women receiving prenatal and postpartum care. Individuals seeking treatment for infertility commonly encounter concrete barriers, such as formalized BMI-based restrictions. These limitations may further compound the isolating and stigmatizing experiences already known to affect women with infertility.

**Methods:**

Qualitative study exploring the experiences and perceptions of patients with larger bodies and infertility. Women seeking fertility care within the past 2 years with a BMI ≥ 30 kg/m^2^ were eligible for participation. Demographic surveys and one-on-one semi-structured interviews were performed. Interviews were deidentified, transcribed, and analyzed inductively using a thematic analysis approach. Major themes and subthemes were identified by two coders with discrepancies being resolved with a third coder.

**Results:**

A total of 33 subjects were included in the analysis. Mean age of participants was 36.9 years. Key themes identified included stigmatizing treatment, perceptions of substandard care, complex body relationships, and quality care as an achievable goal. Many participants reported receiving shaming and judgmental care throughout their healthcare encounters including fertility care, which for several directly influenced their decision to pursue treatment. Participants often reported receiving abbreviated, substandard diagnostic evaluations that in many cases missed critical diagnoses. Participants conveyed significant awareness of the impact of weight on fertility and complex weight cycling histories, and this knowledge paired with these experiences often went unaddressed by providers.

**Conclusion:**

Consistent with the experiences of patients with larger bodies broadly, patients with infertility similarly report receiving stigmatizing treatment and perceived substandard care from their fertility providers. This potential harm to patients is not inevitable; participants report receiving quality care where providers offer humanizing and individualized care. There is a clear need for personalized and patient-centered treatment for this population of patients, that while marginalized, account for an increasing percentage of fertility patients.

## Introduction

Individuals with larger bodies experience a pervasive, resilient form of social stigma that induces psychological and physical harm (1). Furthermore, bias and discrimination against people with higher weights has been demonstrated to independently contribute to increased morbidity and mortality (1). Women with larger bodies report negative interactions with healthcare (2), which may create a barrier to care: there is a direct correlation between the body weight of women and the extent of delay or avoidance of health care (3) including sensitive gynecologic care (4, 5).

Stigmatizing treatment is also highly prevalent among women seeking care during pregnancy. One out of five pregnant and postpartum women report experiencing weight stigma during their care, with Obstetricians as the most commonly-reported source (6). Prior qualitative work examining the narratives of people with larger bodies in pregnancy and postpartum revealed persistent shame and stigma inflicted by medical professionals onto pregnant and postpartum patients (6–11). Where preconception reproductive experiences of people in larger bodies has been explored, patients report routinely being told to leave and return after losing weight, shaming experiences from reproductive providers, and even overt experiences of fertility denial (ex: denying IUD removal) on the basis of weight (7–9).

Infertility affects one in six individuals globally and is associated with significant physical, financial and psychosocial stress. For larger patients, access to fertility care is frequently institutionally restricted by body mass index (BMI). Although there are no standardized restrictions to access to fertility care or IVF in the United States, 65% of surveyed clinics report they enforce BMI cutoffs ranging between 35 and 45 kg/m^2^ (12). Proponents of BMI cutoffs often raise concerns regarding the provision of optimal care for patients with obesity, specifically centered around limitations of transvaginal ultrasound and establishing or maintaining airways in procedures with sedation. However, studies demonstrate that oocyte retrieval can be safely completed in women with higher BMIs in the proper setting (13). Thus, despite data suggesting safety, women with larger bodies in the United States are routinely denied access to fertility care.

Larger patients seeking fertility care face formal BMI-based limitations, and they also face the entrenched weight-based bias and discrimination reported broadly by patients. Furthermore, female infertility is globally associated with both external and internalized stigma that has well-known associations with decreased mental and overall quality of life (14, 15). A recent study explored the perspectives of patients who were unable to access IVF care due to BMI restrictions (16), however, this study was limited to patient feelings towards IVF restrictions. Given the particular vulnerability of women with infertility there is a need to further explore the experiences of women in larger bodies seeking infertility care. To contribute to this body of work and to develop patient-centered solutions, we performed a qualitative study to better understand the lived-experience of women seeking fertility care and explore their experience with diagnosis, treatment, and overall reproductive care.

## Methods

This study was approved by the Institutional Review Board at the University of Michigan. We recruited participants via UMHealthResearch.org, an online platform connecting researchers with individuals interested in research participation, as well as through informational flyers posted in OBGYN and fertility offices, and at time of routine fertility appointments at the University of Michigan Center for Reproductive Medicine. In addition, patients who had been seen in the past 2 years for fertility care, but had not returned for follow up for greater than 6 months were contacted to capture patients who had discontinued fertility care. Patients who met the following criteria were eligible: (1) sought care from a reproductive specialist; (2) >18 years of age; (3) BMI ≥ 30 kg/m^2^; (4) history of infertility or reproductive disorders such as PCOS, diminished ovarian reserve, endometriosis, or desire to utilize donor sperm. Participants were excluded if they experienced infertility but had not seen a fertility specialist.

Participants who met eligibility criteria and agreed to participate completed a baseline demographic survey and underwent semi-structured, in-depth, one-on-one interviews. A research assistant trained in qualitative interview techniques conducted the interviews over zoom, using an interview guide exploring subject feelings regarding their body size, experience seeking fertility care, and reflections on the impact of their body size on the care they received, including how their care may have been different if they were in a smaller body.

Interviews were deidentified, transcribed, and analyzed qualitatively using a thematic analysis approach (17). Initial codes were developed based on a subset of interviews (*n* = 5, 14.3%). All transcripts were coded by two investigators who met at intervals of 5 interviews to discuss emerging broader patterns and differences in interpretation with a third investigator. Related codes were grouped into themes that were reviewed, defined, and finalized based on ongoing analysis of the dataset. Dedoose software was utilized to assist with qualitative data management and thematic analysis. The Standards for Reporting Qualitative Research (SRQR) guidelines were followed for reporting of results (18).

## Results

A total of 71 women were screened with 33 women meeting eligibility criteria and included in the study. Table 1 summarizes the socio-demographic information of the participants. 26 (78.8%) white, 5 (15.2%) African American, and 1 (3.0%) Asian American women participated. 2 (6.1%) participants self-identified as Hispanic or Latina. The mean age of participants was 36.9 years (±4.0 years). All participants completed some type of fertility treatment including oral ovulation induction, injectable ovulation induction, intrauterine insemination (IUI) and/or *in vitro* fertilization (IVF) prior to the interview (Table 2). The most commonly reported fertility treatments were oral ovulation induction (60.6%) and IUI (54.5%). 11 participants (33%) reported IVF.

**Table 1:** Participant demographics.

Characteristics	*n* = 33
Age, years (mean/median)	36.9/37
Race, *n* (%)	
White	26 (78.8)
Black or African American	5 (15.2)
Asian American	1 (3.0)
More than one	1 (3.0)
Ethnicity, *n* (%)	
Non-Hispanic or Latina	29 (87.9)
Hispanic or Latina	2 (6.1)
Other	2 (6.1)
BMI, kg/m^2^(mean/median)	40.9/39.5
BMI, *n* (%)	
Class 1 BMI (30 to <35 kg/m^2^)	7 (21.2)
Class 2 BMI (35 to <40 kg/m^2^)	11 (33.3)
Class 3 BMI (>40 kg/m^2^)	15 (45.5)

**Table 2:** Fertility treatments received by participants.

Treatment	*n* = 33
Oral medication, *n* (%)	20 (60.6)
Intra-Uterine Insemination (IUI), *n* (%)	18 (54.5)
Injectable medication, *n* (%)	15 (45.5)
*In Vitro* Fertilization (IVF), *n* (%)	11 (33.3)
Other, *n* (%)	6 (18.2)
None	0

Table 3 shows major themes and subthemes identified. Participants' narratives broadly revealed stigma in treatment, complex body relationships related to body size and fertility, and discrepancies in care with many women perceiving substandard care and others experiencing high-quality care.

**Table 3:** Major themes and subthemes.

Themes and subthemes
Stigma, bias and judgement
Direct stigmatizing interactions with providers
Intersecting minoritized identities
Anticipated stigma and internalized bias
Avoidance of care
Substandard care
Abbreviated diagnostic evaluations
Delay in diagnosis
Limited fertility treatment
Fractured healthcare experience
Intersection of body size and infertility
Prior experiences with weight and infertility impacting self-worth
Patient knowledge regarding weight
Provider lack of acknowledgement of patients' struggles
Quality care is possible
Humanizing care
Positive experiences with weight related guidance
Patient-guided solutions

### Stigma, bias, and judgement: “You don’t treat your skinny patients like this” (ID 16)

#### Stigma in fertility care

Participants reported stigmatizing interactions with their care team related to their weight throughout their fertility experience. Many participants felt that their providers treated them with diminished compassion due to their body size. Participants often expressed feeling that their providers saw them as lesser for their weight. One patient reflected, “I was automatically like pinned as, oh well, she's overweight, so either she…doesn't have good self-control or she doesn't have the education” (ID 35). Another participant said, “You go to these doctors for help, and…[instead] I felt like such a burden… I hated that these people looked at me like I was just this big, fat, lazy slob” (ID 61). Many participants described being made to feel “subhuman” in their interactions with providers:

“I think society in general treats overweight people differently. Not just doctors, but doctors are the ones that hurt the most when you're trying to have a baby…I'm sick of doctors… I'm sick of them not treating me like I'm a human.” (ID 61)

“And so when it's just kind of like, we pull this lever, we pull this lever, and boom, bang, you're pregnant. I think when you remove the empathy from the situation, you just start to feel like. very bovine. Am I a cow? It's just…[I wish I had been] treated a little more humanely, and with a little more humility to just kind of have respect for your body no matter what shape it is” (ID 41).

In these examples, participants characterize the dehumanizing treatment they underwent as directly related to the perceptions and assumptions healthcare teams hold against people with a larger body size.

#### Intersecting minoritized identities

Weight-related stigmatizing experiences in fertility care often overlapped with stigma relating to additional intersecting marginalized identities of participants including race, ethnicity, sexuality, class, and age. An LGBTQ+ participant shared frustrations with insensitive care that culminated in her partner being asked to carry the pregnancy:

“All throughout the appointment they kept talking to [my partner] and trying to convince her to be open to fertility care, asking her a lot of questions. And she was like, ‘Look, I'm not interested.’ And there were comments about my weight compared to hers, that made me… feel like they were right off the bat assuming that I wouldn't be able to get pregnant because I was overweight. I had an overweight BMI and she did not. So that was upsetting…. She's non-binary. She doesn't identify with femininity, and she can't see herself as being pregnant.. [so it all] induces some level of dysphoria too.” (ID 47)

This participant's obesity interacted with her minoritized status as having a non-binary partner who was assigned female at birth: the care team made assumptions not only about the participant's fertility but also about her partners' fertility, gender identity, and reproductive choices, decreasing the care they received even further. Another participant shared how her age and perceptions of her economic status contributed to her treatment experience:

“I kind of do [feel like my healthcare provider team treated me differently than other patients because of my body size]. And then at the time, I didn't know if it was my age also, because I was 23 at the time.. I feel like there's this perception that like, almost like weight goes along with poverty…[They think,] ‘you're young, and you're overweight, [and you're poor]; you must not be ready to have a baby'” (ID 56).

For this participant, her healthcare providers' negative interpretation of her age, class, and weight compounded one another, and the participant was left feeling that they did not find her worthy to become a parent.

One Latina participant explained how the overlap of weight bias and racism was front of mind for her in fertility care encounters:

“As a plus-size woman as a woman of color, I think we carry a lot of baggage going into our medical encounters… A lot of people have stereotypes about fat people and not being clean and being lazy and as a Latina, like they call it dirty Mexican. So, I'm just like I always made it a point to go and take a shower right before my [appointments]. But I'm thinking, oh, I didn't shave my legs, or I didn't do this, or I didn't do that. Like we know as plus-size people we're being judged.. Culturally, like it can be challenging to show your reproductive parts to somebody. But then on top of that like being plus-size and I remember them having to take out the speculum and take another speculum and you're just like, oh God would this be happening if I wasn't fat? … It just makes those encounters so much more stressful.” (ID 33)

One participant explained that the stress of these encounters and feeling that her providers dismissed her symptoms, specifically due to the interaction of anti-fat bias and racism, impacted her approach to provider interactions: “You kind of shut down and you don't necessarily share all the information or have the best interaction because now you have your guard up.” (ID 33).

#### Anticipated stigma and internalized bias

Constant vigilance was very commonly reported by participants: even when they did not describe overtly discriminatory treatment in fertility care, many communicated anticipating biased treatment. Participants were primed to anticipate discrimination from prior stigmatizing experiences within other healthcare fields. Stories of weight-related mistreatment among peers also played a role in priming patients to anticipate negative treatment. Expecting biased care took a significant toll on participants in their fertility journey:

“I remember when I was a master's student…one of the doctors was giving us a tour of like the gynecology area or something, and he showed us a table and a scope and then he was like, yeah, this is the one we use for the really fat ones. You never know what goes on because of that experience. There's been always that kind of wondering like, are they saying something about me?” (ID 33)

“I'm 39, I've accumulated bad experiences with healthcare systems. So even a neutral experience … is anxiety producing and bad a lot of the times.. I'm kind of on a high alert in a medical setting for like, okay, when is it gonna happen? When are they gonna say something or do something that's gonna be harmful or bring up a past issue?. I'd go into those experiences with a new provider expecting it to potentially be really bad. Expecting them to blame me for the problems I'm experiencing, expecting them to maybe make a recommendation that I know is damaging” (ID 71).

This participant reports significant fear and anxiety as she braces for stigmatizing treatment in each encounter with medical care as she has been subjected to blame and damaging recommendations in the past. This anticipation of negative treatment based on prior experiences led others to avoid and or delay seeking fertility care:

“I'm really upset with myself that I put it off for so long because of my weight…I was afraid to do it because I was afraid of going to the doctor and being embarrassed by my weight. Because that's what happens when you go to the doctor, you go in for a cough and they say, ‘Well, you need to lose weight’ or you go in and say, ‘I broke my arm.’ ‘Oh, you should probably lose some weight and maybe you won't.’ So I was just so afraid to go on this journey and be told that” (ID 23).

This participant reports expecting physicians to blame her body size for all medical ailments based on prior experiences, and she delayed seeking fertility to avoid further shaming experiences. Ultimately, though, this quote reveals the participant self-blame: the participant is “upset with herself,” rather than her medical teams, for the barriers she experienced to seeking out care.

### Perceived substandard care: “They don't want to get to the bottom of it. They just say, ‘Oh, it's your weight’”(ID 67)

#### Insufficient evaluations

Participants often reported receiving substandard fertility care, including abbreviated diagnostic evaluations and treatments limited to nonspecific suggestions to lose weight. Many participants felt that their infertility was entirely attributed by their providers to their weight, and they did not receive the thorough evaluations afforded to patients with smaller bodies.

“I asked [my doctor], ‘Can we look at my tubes? I read that maybe my tubes could be blocked and that could be contributing to it, or even after the testing of tubes, looking at them, it could promote fertility. I read that too.’ And he basically said no it wouldn't matter either way” (ID 37).

In this instance, the participant was denied a standard component of the fertility evaluation. Instead, the subject was swiftly dismissed and the provider advised weight loss without affording this subject the standard of care. This substandard care as evidenced by general dismissal and lack of basic evaluation was commonly noted by participants:

“Instead of doing tests first, they immediately went to age and weight, and I was like, okay, but you haven't even seen me in person, you just looked at what my stats were, and you based your opinion on that” (ID 35).

In addition to lack of diagnostic evaluation and the assumption of weight as the sole-cause of infertility, assumptions were also made about the presence of other health conditions. For example, healthy subjects were assumed to have diabetes or hypertension, and one participant was put on metformin despite not having PCOS or insulin resistance (ID 56).

#### Delay in diagnosis

Ultimately, insufficient diagnostic evaluations led to providers missing significant fertility diagnoses, including hypothyroidism and PCOS. A participant reflected on the delay of diagnosis, concluding that her provider “just sort of saw a fat person and thought that was the problem and didn't even bother to diagnose this very basic thing that she should have known about” (ID 71). One patient did not receive her diagnosis of antiphospholipid syndrome until visiting her third fertility provider:

“I haven’t been given a reason why I couldn't conceive. He [the doctor] did an ultrasound on me he looked at my weight and he just flat out told me right there that basically I'm too fat and he won't do anything for me…He said my BMI was too high and there was nothing he could do…When I came to [Doctor Name], it was different because, one she listened to everything I said and I think literally in seven years, it's the first time I feel like someone was really listening to me and not judging me. And she put me on medicine for my thyroid. No doctor in the last seven years has addressed that. She found out I have a blood clotting disorder that no one's really addressed. So there's like several factors that have been discovered about myself outside of being overweight that are causing my miscarriages.” (61)

In this case provider assumptions and delay of diagnostic evaluation led to considerable delay in the diagnosis of a treatable disease with significant maternal and fetal burden.

#### Limited fertility treatment

Participants reported limitations of treatment secondary to their body size. Many participants described being turned away from care: “They [said], ‘You have to lose weight.’ So there was no leeway, it was you have to lose weight in order to be able..to do fertility care” (ID 59). Another participant was told, “see if you can lose some weight and then come back” (ID 10). Some participants specifically cited BMI limitations held by clinics, and others named specific procedures (egg retrievals, IVF) that were unavailable to them due to their weight.

Ultimately, participants felt these limitations and delays contributed to negative fertility outcomes.

“I feel like it's already a very high stress situation. And just adding that on the whole like, okay, so now you have to lose weight in order to be able to do this. It's just an added stressor. … When you're older, you don't have time to be wasting a year trying to lose weight, it's like you got to be able to dive right into it” (ID 59).

The delays in fertility care compound the high-stress baseline experience of infertility especially among women of older reproductive age. In this case, by being required to first lose weight, her fertility care was delayed—a delay that, based on her age, has significant negative impacts on her possibility of a positive fertility outcome.

#### Fractured healthcare experience

Many participants transferred care between fertility providers. Participants reported changing care when their initial provider would not offer them any interventions until they had lost weight (ID 30) or would not offer specific interventions, like IUI (ID 18) or IVF (ID 10). Multiple participants sought care out of state for the cheaper price and reputation for fewer weight related restrictions (ID 18, 16). Other participants transferred care after directly stigmatizing interactions with their provider, such as a participant who was pressured to lose weight while the provider performed a sensitive exam (ID 47), and others transferred after more subtly subpar care, including feeling that they were not taken seriously (ID 35). Often, these transfers represent a form of patient self-advocacy to be able to receive non-stigmatizing care and specific desired fertility treatments. One participant explained:

“After I believe it was four miscarriages, OB did some testing, couldn't find anything, actually sent my husband down to [Institution] to talk to a genetic counselor. They had us register both of us but they wouldn't test him…So we tried again, had a couple more miscarriages and then I learned what a reproductive endocrinologist was…after eight losses. I found [a reproductive endocrinologist] downstate, ‘cause I did some research [on a local clinic] and [learned] ‘lose weight’ [is] like their mantra.. So I found [a different] one a couple hours away. We went there. They ran a karyotype test on my husband, discovered he had a balanced translocation, so that led us to IVF…Then we switched clinics to [a different state], because they were cheaper. And we did three more rounds there.. and then we pursued embryo donations and I switched to a third clinic that was local.. Again [I] did research on ones that had BMI limits, things like that. [With the third clinic,] I had a successful pregnancy and then I'm actually currently pregnant again” (ID 16).

This subject describes burdensome and excessive research as well as significant travel required just to find clinics that would offer a thorough diagnostic evaluation and treatment. Ultimately this resulted in the need to see at least 3 different fertility providers. Thus, while a savvy patient may be able to navigate the significant hurdles placed by body size it may often involve frequent transfers of care that can contribute to a fractured care experience.

### Intersection of body size and infertility: “You feel like your body is broken” (ID 25)

#### Prior experiences with weight and infertility impacting self-worth

Participants detailed complex relationships with their own body size that were not often considered by their providers. Most patients expressed a lifelong “struggle” and “battle” with their weight: “I feel that my whole life [my weight has] been a struggle, it's never been just like certain times of my life, it's been my entire life” (ID 35). When asked about their feelings on their current weight, participants gave a range of responses, with some responding very negatively “Oh, terrible” (ID 43), others expressing some ambivalence “I'm trying to lose weight, but I am sort of okay with where I'm where I'm at” (ID 49), and others expressing acceptance and positivity of their size after a lifetime of fluctuation:

“At this current moment right now, I feel okay [about my weight]… It's up and down. There are times when I truly hate my body.and then there are other times when I feel very, more at peace. Like, this is who I am, and this is how I look, and my body produced people, so that's pretty cool.” (ID 17)

“I've been overweight pretty much my entire life, I kind of just reached a point where I was like, yeah… It's really hard for me to be motivated to lose weight when I am this hot.” (ID 41)

Many participants detailed an extensive history of prior weight loss attempts including many types of diets, commercial weight loss programs, stimulants, and exercise programs. Many also had engaged in disordered eating at some point in their lives: “I know how to eat right. I also, to be completely honest, know how to starve myself” (ID 37). Infertility further complicated body image for many participants: “It's already emotional enough to do any type of fertility care because you feel like your body is broken… but adding like your body is broken and can't metabolize food in a way that society feels is perfect or normal or any of those things is really hard” (ID 25).

#### Patient expectations regarding weight

Most participants reported having some expectation that higher weight negatively impacted reproduction prior to being told by a provider, either from doing their own research, from family members and friends, from prior diagnoses such as PCOS, or from a sense that obesity impacts all aspects of health. Participants also had an understanding that there are many complex causes of infertility, and they sensed that their providers were withholding this nuance by blaming their fertility solely on their weight:

“Well, I just kind of feel that if it was just, if body size was …the biggest factor, then there would be no skinny women in the fertility clinic. But that's not the case because I have gone to the fertility clinic many a times and there are women of all colors, shapes, and sizes. And that's, to me, the bottom line. I don't know what the emphasis is on my weight when there are… there are women who are very slender who also have unexplained fertility. Sometimes I feel like it's kind of a cop out, like [my fertility providers] don't really know.” (ID 10)

Participants mentioned noticing people with higher weights who had become pregnant, becoming pregnant themselves at higher weights than they were at currently, and noticing people with smaller bodies experienced infertility as well. Conversations with providers often failed to acknowledge the evidence of the complexity of infertility they had seen and lived.

#### Provider lack of acknowledgement of patients' struggles

In discussions about weight loss related to their fertility care, providers often ignored participants' lived experiences and complex understanding of weight. Participants felt their providers made assumptions about their health behaviors:

“[They assume] I must be eating terrible. I must not be doing anything.” (ID 52)

“I think when people see overweight women, they just assume we're lazy, which I am not and many of us aren't. I work hard. I'm trying to lose weight.” (ID 61)

“[My provider told me,] ‘If you just stop drinking soda, you'd probably drop 10 pounds.’ Okay, I don't drink soda… That's not the reason I'm fat…Why did you make that assumption?” (ID 41)

In turn, providers often offered participants nonspecific, limited, unhelpful advice about weight loss. Some participants reported being told to “just lose weight” without support through the process.

“It's always the thing of, ‘You need to be thinking about your weight’ [but we’re] never given resources…it was always a [change] your weight type of thing, but not helpful.” (ID 67)

Others were vaguely advised to diet and exercise:

“Diet and exercise, go run a marathon, I guess, and eat salad for the next three months. Yeah, good luck…[They tell me], ‘Well, lose weight.’ [I ask],’How do I lose weight?’ [They say,] ‘Go home and count your calories and restrict your food and eat less than normal and go to the gym five days a week. Okay, good luck. Come back in three months.’” (ID 49)

“[Providers have told me] ‘diet and exercise,’ every single time. ‘Count your calories.’ ‘Lower calories, more exercise.’ Every time.” (ID 35)

“‘Eat better, exercise.’ I've never, like, they've never given me a plan. They've never said, ‘let's come up with something,’ it's just, ‘eat less carbs and walk’. And that's what's really frustrating. .. [I'm] always told, ‘eat healthier and exercise and you'll lose all of this weight magically.’” (ID 23)

Others report being told by providers to follow fad diets, like eating only shakes, salads with lemon and pepper (ID 71), chicken broth only; “there's always a new diet they want you to follow” (ID 70). Participants wished that providers took a more careful approach that respected the knowledge patients had about their own bodies.

One participant stated she wished providers had “a basic understanding of diet culture and respect for the intelligence of people who are fat…I think probably it's a fair assumption that most people who are 39 and coming into their second round of IVF and they have PCOS… Probably, they know some stuff. They've done some research on vegetables. They know what a salad is..Most of the medical providers that I've had that have not felt respectful to me… [just] assume that I'm just lazy or dumb. And so [I wish providers had] a willingness to wrestle with that assumption.” (ID 71).

### Quality care is possible: “They knew me by name, they knew my whole history. I wasn't just a patient.” (ID 70)

Conversely, several participants highlighted the possibility of obtaining high-quality, respectful care.

#### Empathetic, humanizing care

Participants noted several elements in their care interactions that created for a positive experience. These included a care environment that was welcoming and encouraging; the opportunity to participate in (and guide) care through shared decision making; providers addressing fertility issues that were unrelated to their body size; and often, the absence of focus on weight and weight loss was in itself positive.

“So, the positive experience was, like I said, really feeling like it was patient centered… I was able to meet with people, discuss options. I was presented with options the second time around, which was nice. ‘Let's try this, let's try this. If this doesn't work, then we can go to this.’ And when I asked, ‘Can we look at my ovaries? Can we look and see if my tubes are blocked?’ I wasn't blown off. They were like, ‘Yeah. We can look at all these things.’ …[I was being] listened to and being taken seriously…[After my] failed IUI experience, [my doctor asked,] 'Should we move towards the next step?’ And I asked them, ‘Can we just try one more time, and can we be a little bit more aggressive with the medication, maybe try to get two follicles instead of one, maybe that would help?’ And she was like, ‘Yeah. Let's try it. Let's go for it.’ … And that's when I got pregnant. So and that was really a good experience.” (ID 37)

Participants also had positive experiences where they felt their weight was not the only component of their fertility that was addressed:

“And like I said, when I went to [First Doctor Name] office, they were great. They made sure all of my medical stuff, they ran all the proper tests, just the same as everything that the university's doing now. ‘cause I did ask [First Doctor Name] office, I was like, ‘Is my weight gonna be a problem?’ They were like, ‘No, we just have to adjust some things.’ But they were the ones that were like, ‘Just because you're overweight doesn't mean you can't carry a baby or two. You could carry a baby full-term and be just fine.’ I'm like, ‘Well, yeah, there's tons of people out there that are over 120 pounds and they're able to carry babies and work and do all this other stuff.’” (ID 57)

“I didn't feel like she was looking at me like I was a fat piece of crap, which is what I felt like most of the time. She thoroughly went through my blood work, which is something that no one's done. She had a plan for me and she just talked to me. She let me be the crybaby that I was and she didn't judge me. .. **And I felt like a person for the first time** (emphasis by authors) in seven years, I just wanted to have a baby, you know? [She] was the first person in a long time who made me feel like maybe I have a chance of being a mom.” (ID 61)

This participant describes this interaction as both humanizing—she felt like a person for the first time in years—and encouraging.

#### Patient guided solutions

Participants were asked what advice they would give other patients with obesity and infertility; many participants recommended advocating for oneself and transferring care away from clinics where they felt disrespected. Participants also gave advice for navigating and avoiding stigma and substandard care, including provider assumptions about patient behaviors, dismissiveness, and misalignment of provider and patient goals:

“[Remember that] you know your body more than anyone else knows your body. And so being able to advocate for yourself, when something doesn't feel right, or you want to explore something further, that it's okay to be firm … And it's okay to then find a new doctor, there's lots of doctors out there, and there's going to be physicians that want to help those of us that are going through it.” (ID 49)

“Get second opinions..and look for a doctor who you feel comfortable with. If you are referred to someone and.. you don't have that good feeling in your stomach, that they're like, ‘Okay. This person's really going to help me,’ try to find someone else.. Don't just go with the first option in the hopes that it works, find someone that you feel comfortable with and you're confident with.” (ID 37)

#### Recommendations to providers

Participants asked providers to see them as a whole people, not just their weight and not just as numbers. They ask for a more careful approach to assessing history and the overall state of health. As stated by one participant:

“I would like them to know that my weight does not define me… I was immediately looked at, as all of these alarms because of my weight. And I just really wish they would take the time to get to know the patient and get to know their medical history vs. just seeing a number and then writing them off and giving them all of these warnings because maybe had they talked to me, and said, oh, you know what, she has no conditions other than being overweight that are really putting her at risk. So, that's the biggest thing to me, is just get to know your patients other than their weight. There is so much more into their medical life than just that.” (ID 23)

## Discussion

In this study, we describe the experiences of women with larger bodies and infertility navigating fertility care and treatment. Participants often reported feeling that they were treated with diminished respect and compassion due to their body size, which for some participants compounded with other intersecting minoritized identities including ethnicity and sexuality.

Anticipating stigma was frequently mentioned by participants as a contributor to stressful fertility encounters and as a reason to delay seeking fertility care. Additionally, many participants experienced incomplete diagnostic evaluations and limited fertility treatment options contributing to delayed diagnoses, delays in receiving treatment, and frequent transfers of care. Participants described complex relationships with their own bodies related to weight and infertility which were frequently disregarded by fertility providers' advice to “just lose weight.” Positive fertility care was reported by participants where their providers shared hope regarding their capacity to become pregnant and considered factors in their fertility aside from solely their weight.

### Stigma and healthcare avoidance

In a recent large systematic review and qualitative synthesis of over 32 qualitative studies exploring the experiences of patients with larger bodies, one of the most powerful constructs/themes related to stigma, judgement, shame, and blame (30). In addition to experiences with generalized social stigma, patients have reported negative treatment directly from their healthcare team ranging from lack of respect and compassion to more frank breaches of dignity (30). Consistent with prior qualitative studies of pregnant and postpartum women with larger bodies (6–10), our study found that the shame, stigma, and judgment that characterizes pregnancy in this population begins even earlier in people who seek fertility care and is often inflicted by medical professionals.

In the current study, participants reported anticipating incomplete care and demeaning treatment by their fertility clinicians due to their weight, even when they could not recall overt evidence of weight bias in their experiences; this worry was often founded in participants' prior stigmatizing experiences in healthcare. There exists a significant emotional toll of bracing for mistreatment: in an experimental study of 146 women with higher weights, participants who were tasked to interact with a neutral-acting peer that participants had been told held an explicitly anti-fat attitude (vs. no bias) were found to experience poorer cognitive performance, lower self-esteem, increased negative emotions, and increased rumination with negative effects increasing with increasing weight (19). In the current study, participants reported a range of mechanisms to try to avoid stigma and judgement for their weight, including delaying and avoiding fertility care. Delay of healthcare for fear of mistreatment has similarly been demonstrated among a population of low-income women with higher weights who reported delaying or avoiding healthcare visits due to the expectation that they their lives would be reduced to weight-based stereotypes in their clinical encounters (20). The current study highlights that healthcare delay and avoidance due to prior stigmatizing experiences extends to patients with obesity seeking fertility care, which may have significant implications for patients' ultimate success with fertility interventions.

### Substandard care

Participants frequently described abbreviated diagnostic evaluations that relied on provider assumptions about patients with obesity rather than participants' actual health status. Fertility treatments offered were limited with participants often being told to “lose weight and come back” or being unable to access treatments due to formal BMI limitations; it was challenging to assess where participants faced formal vs. informal limits in our study. A recent study investigating patient perceptions of BMI restrictions on fertility care described formal BMI limits as frustrating and discriminatory, especially where these limitations were not accompanied with explanation and many participants felt BMI limits denied parenthood on one singular factor, rather than overall picture of health (16).

Another component of participants' experiences included transfers of care, often due to stigmatizing interactions and limited treatment options. These fractured care experiences create delays, gaps in care, incomplete patient health histories. This parallels findings of a qualitative study of 15 patients with obesity and endometrial cancer in which participants described frequently transferring providers to be able to find those who would offer treatment (21), and a study surveying 501 pregnant and postpartum women found that 7% reported changing providers due to negative treatment; those who reported transferring care had a significantly higher mean pre-pregnancy BMI than those who did not (6). Patients with larger bodies seeking fertility care are at risk for discontinuous care experiences, which may ultimately impact outcomes due to substandard, interrupted care.

### Intersection of body size, other minoritized identities, and infertility

Participants revealed complex relationships with their own bodies as related to size and infertility. People with obesity often struggle to view their bodies positively, often using negative language to describe their physical appearance and describing feelings of guilt, shame, and blame associated with their weight (22). In a recent qualitative study of Italian women undergoing treatment for infertility, participants shared how the process of receiving infertility treatment made them feel that their bodies were “devastated” and “worsened” due to the therapies and physical side effects (23). The current study supports this interplay of infertility, weight, and fertility treatment that can compound to lower patients' body image and self-worth and ultimately lead to the feeling of one's body being “broken”. Participants in the current study also shared extensive weight loss, weight cycling, and disordered eating histories. Disordered eating is more prevalent among those with obesity and a recent systemic review reveals rates of current or past eating disorders are also higher among women seeking fertility treatment than the general population (24–26).

Despite these histories and complex narratives, participants often encountered fertility providers who failed to inquire about patients' prior weight loss attempts and offered very limited and non-specific advice or instead offered potentially dangerous recommendations especially to those with a history of disordered eating. Participants widely reported clinicians relying on their assumptions about patient with obesity's health behaviors. Prior studies have shown physicians to hold explicit and implicit negative attitudes about patients with obesity (27). In the current study, clinicians' reductive approaches erode the patient-provider relationship by further stigmatizing patients. Fertility providers instead should approach their patients with curiosity, and they should not be surprised that their patients may have extensive prior experiences with weight loss, weight cycling, or disordered eating. Clinicians should anticipate that their patients may already have significant knowledge and awareness regarding the intersection of body size and reproduction, which may be contributing to negative body image and self-blame for infertility.

### Quality care is possible

Many participants mentioned fertility clinicians that never acknowledged their higher weight; these participants reacted with relief and gratitude. This contrasts with others where weight was used as a reason to withhold and delay care, offer futile advice, or blame patients for their infertility. There exists little research examining fertility clinicians' perspectives toward treating patients larger bodies, but an integrative review of communication practices of obstetric healthcare professionals suggest that providers may be hesitant to broach the topic of weight due to concerns of stigmatizing patients, fear of instilling a sense of guilt in patients, and feelings of discomfort (28). Where providers are aware of the weight bias their patients have experienced, they might be less likely to address weight. A recent review examining fat shame and blame in reproductive care suggests that weight bias training be implanted through professional development and be required education (Ward and McPhail). The authors also suggest that if health care providers believe that reproductive risks must be communicated to larger women that providers focus on positive aspects of pregnancies and opportunities for positive health promotion as well as a focus on health and not weight-centered approaches (Ward and McPhail). In our study, we found that positive experiences with fertility-driven metabolic and weight loss counseling often addressed several other components of fertility, not just weight and considered patients' histories in developing treatment.

### Strengths and limitations

Strengths of this study include the diverse range of fertility treatments completed by participants. To our knowledge there is only one prior study that specifically looked at larger women with infertility and was limited to women denied fertility treatment secondary to BMI restrictions (16). The current study is able to detail the broader patient experiences seeking and receiving a range of treatments in a range of contexts. Another major strength of this study its qualitative nature. The detailed, expansive narratives of larger patients undergoing fertility treatment must be heard to guide patient-oriented solutions to improving care for this marginalized group.

The results of this study should also be considered in the context of several limitations. First, this study did use BMI in the inclusion criteria. There is significant literature highlighting the limitations and potential harms of BMI including its poor individual risk prognosis, lack of accuracy based on sex and race, and historical harm (29). The use of BMI was included in this study to capture the experiences of individuals who did and did not have access to care based on BMI restrictions, which are commonly employed. Second, the current study did not formally collect information regarding the success of weight loss attempts or of fertility treatments. This study may also be limited in that people with greater comfort and experience discussing body size were likely overrepresented in the study group, and they may be overrepresented in the analysis given the length of their responses.

## Conclusions

In summary, larger women encounter significant challenges pursuing and receiving fertility care. Consistent with the experiences of patients broadly, patients with larger bodies and infertility are subjected to stigmatizing interactions, limited diagnostic and treatment options, and substandard care. Some unique aspects of experiences within infertility care include delays in care related to formal and informal weight-based restrictions in care in many cases leading to higher maternal age at presentation and the complex interplay of fertility, fertility treatments, and weight on patient self-image. It is imperative that fertility care providers understand that their patients have had negative prior experiences with weight bias in medical care, and they should seek to examine and overcome their own biases and assumptions. This study also shows that this harm to patients is not inevitable; participants shared experiences of trusting relationships with their clinicians and receiving comprehensive fertility care that left them feeling empowered and respected. Further research is needed to determine successful approaches to providing quality care for this population.

## Data Availability

The raw data supporting the conclusions of this article will be made available by the authors, without undue reservation.
